# Room temperature amine sensors enabled by sidewall functionalization of single-walled carbon nanotubes†

**DOI:** 10.1039/c7ra13304a

**Published:** 2018-02-01

**Authors:** Clara Paoletti, Maggie He, Pietro Salvo, Bernardo Melai, Nicola Calisi, Matteo Mannini, Brunetto Cortigiani, Francesca G. Bellagambi, Timothy M. Swager, Fabio Di Francesco, Andrea Pucci

**Affiliations:** Department of Chemistry and Industrial Chemistry, University of Pisa Via G. Moruzzi 13 56124 Pisa Italy fabio.difrancesco@unipi.it andrea.pucci@unipi.it; Department of Chemistry, Institute for Soldier Nanotechnologies, Massachusetts Institute of Technology 77 Massachusetts Avenue Cambridge MA 02139 USA; Institute of Clinical Physiology, National Council of Research (IFC-CNR) Via G. Moruzzi 1 Pisa 56124 Italy; Department of Chemistry “U. Schiff”, University of Florence Via della Lastruccia 3-13, 50019 Sesto Fiorentino (FI) Italy; National Interuniversity Consortium of Materials Science and Technology (INSTM) Via G. Giusti 9 50121 Firenze Italy

## Abstract

A new series of sidewall modified single-walled carbon nanotubes (SWCNTs) with perfluorophenyl molecules bearing carboxylic acid or methyl ester moieties are herein reported. Pristine and functionalized SWCNTs (p-SWCNTs and f-SWCNTs, respectively) were characterized by X-ray photoelectron spectroscopy (XPS), Raman spectroscopy and scanning electron microscopy (SEM). The nitrene-based functionalization provided intact SWCNTs with methyl 4-azido-2,3,5,6-tetrafluorobenzoate (SWCNT-N-C_6_F_4_CO_2_CH_3_) and 4-azido-2,3,5,6-tetrafluorobenzoic acid (SWCNT-N-C_6_F_4_CO_2_H) attached every 213 and 109 carbon atoms, respectively. Notably, SWCNT-N-C_6_F_4_CO_2_H was sensitive in terms of the percentage of conductance variation from 5 to 40 ppm of ammonia (NH_3_) and trimethylamine (TMA) with a two-fold higher variation of conductance compared to p-SWCNTs at 40 ppm. The sensors are highly sensitive to NH_3_ and TMA as they showed very low responses (0.1%) toward 200 ppm of volatile organic compounds (VOCs) containing various functional groups representative of different classes of analytes such as benzene, tetrahydrofurane (THF), hexane, ethyl acetate (AcOEt), ethanol, acetonitrile (CH_3_CN), acetone and chloroform (CHCl_3_). Our system is a promising candidate for the realization of single-use chemiresistive sensors for the detection of threshold crossing by low concentrations of gaseous NH_3_ and TMA at room temperature.

## Abstract 2

Chemiresistive sensor based on sidewall modified SWCNTs with perfluorophenyl molecules bearing carboxylic acid were prepared for the detection of low concentrations (5–40 ppm) of gaseous NH_3_ and TMA at room temperature.

## Graphical abstract

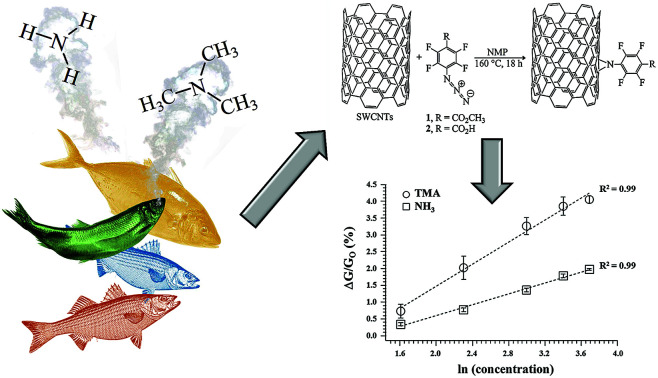

## Introduction

There has been an explosion of interest in carbon nanomaterials over the last 30 years. Carbon nanotubes (CNTs) have attracted a great deal of this attention because of their outstanding electrical, thermal, and mechanical properties as well as their high aspect ratio.^1–4^ Because of their delocalized electronic structure and the accessibility of their π-electronic states to external perturbations, the electrical properties of CNTs are also sensitive to changes in the local environment.^5–9^ This intrinsic property has been widely exploited to realize CNT-based sensors for different external stimuli, especially gases and vapours. Moreover, the electrical nature of these responses allows for facile integration into other platforms such as the resonant circuits of a quartz crystal microbalance (QCM) or in a commercial RFID tag^10^ where a network analyser or a mobile phone^5^ can be used as the reader.

In particular, the detection of amines is important for industrial/environmental monitoring,^11^ food quality control^12^ and disease diagnosis.^13^ For example, in food quality control, the levels of ammonia (NH_3_), trimethylamine (TMA), dimethylamine and triethylamine can be used to assess the spoilage of fish.^14^ Devices capable of monitoring NH_3_ and TMA would enable quality validation throughout the food chain and identify those responsible for incorrect preservation. More specifically, fish is fresh if TMA remains below 10 ppm, whereas a concentration between 10 ppm and 50 ppm indicates preliminary rot, and above 60 ppm fish is considered rotten.^15^

To date, several studies have been performed concerning the development of chemical sensors sensitive to NH_3_ and TMA. Most of them involved the use of metal oxides working at temperatures above 200 °C.^16,17^ Recently, researchers have started combining the properties of metal-oxides and carbon nanomaterials, for example by decorating the surface of CNTs or graphene with metal-oxides nanoparticles.^18^ However, good results were only obtained at high temperatures and/or high concentrations of analyte.^19^ Notably, pristine carbon nanotubes (p-CNTs) change their conductivity upon the interaction with gas molecules,^7^ but the sensitivity and selectivity towards gas analytes are poor. Covalent or non-covalent modification of CNTs is an accessible procedure to provide graphitic materials with modulated functionalities and potential sensor response.^6,20,21^ Among the possible covalent functionalizations of CNTs, nitrene chemistry has proved to be an effective strategy under mild conditions being also useful for potential scale-up synthesis.^22,23^

In this work, we exploit the nitrene chemistry for the introduction on the SWCNTs surface of aziridinyl moieties that are able to provide an effective sensing response towards gaseous NH_3_ and TMA at room temperature. Notably, the degree of SWCNTs functionalization was determined by X-ray photoelectron spectroscopy (XPS), whereas Raman spectroscopy and scanning electron microscopy (SEM) assessed the structural integrity of CNTs after functionalization.

## Experimental

### Chemicals and instrumentation

SWCNTs were obtained from Nano-C Corp. (ultra-purified SWCNT, UPT200) and used without further purification. Methyl pentafluorobenzoate (99% purity) was purchased from Sigma-Aldrich and used as received. Chromium (purity 99.99%) and gold (purity 99.99%) were purchased from R.D. Mathis. All chemicals were purchased from Sigma-Aldrich and used without further purification.

XPS analysis were performed as described by Salvo *et al.*^24^

^1^H and ^19^F NMR data were recorded on a Bruker AVANCE III HD 400 instrument at 400 MHz and 376 MHz, respectively. Chemical shifts are reported as *δ* values (ppm) and referenced to the residual protons of deuterated CDCl_3_. High resolution mass spectra were measured with a Bruker Daltonics APEXIV 4.7 Tesla FT-ICR-MS using ESI ionization.

Raman spectra were measured by a Horiba Jobin-Yvon LabRam (HR 800) Raman Confocal Microscope, with a laser excitation at 532 nm and a laser spot size of 1.2 μm. The Raman band peaks were calculated *via* Lorentzian curve fitting by the Levenberg–Marquardt algorithm.

Functionalized CNTs were characterized by SEM using a JEOL JSM-6700F field emission SEM (FESEM). CNTs analysis was performed using the public domain Image Tool 3.00 version image analyser program developed at the University of Texas Health Science Center in San Antonio and is available on Internet at http://ddsdx.uthscsa.edu/dig/itdesc.html.

An EmStat-MUX handheld potentiostat (PalmSens Instruments) was used to determine conductivity values from the sensor array.

A Fluke 287 True RMS (Fluke Corporation) was used as digital multimeter. Digital mass flow controllers (MFC) were from Sierra Instruments. A KINTEK gas generator system was used for gaseous VOCs detection measurements. Relative humidity was measured using a humidity meter (Extech).

The syntheses of methyl 4-azido-2,3,5,6-tetrafluorobenzoate (1) and of methyl 4-azido-2,3,5,6-tetrafluorobenzoic acid (2) were reported in the ESI.†

### Preparation and characterization of functionalized SWCNTs

As an example, an aliquot (20 mg) of SWCNTs was placed in a 100 mL Schlenk flask and dispersed in 20 mL of *N*-methyl-2-pyrrolidone (NMP). The mixture was sonicated for 2 h. The Schlenk flask was then equipped with a condenser and the suspension was bubbled with argon for 30 min. An aliquot of 200 mg of (1) was added to the mixture, which was then heated to 160 °C and left under argon atmosphere and constant stirring for 18 h. The mixture was cooled at room temperature and the product was isolated by precipitation in acetone. The solid was recovered by centrifugation at 14 000 rpm for 20 min. The separated solid was re-dispersed in CHCl_3_ with the aid of ultrasonication and then recovered by centrifugation at 13 000 rpm for 15 min. The purification process was repeated four times. The final black solid (SWCNT-N-C_6_F_4_CO_2_CH_3_) was dried under vacuum at 70 °C overnight. An identical procedure was followed for the preparation of SWCNT-N-C_6_F_4_CO_2_H, by using 2.

Before the XPS analysis, some drops of analytical grade dichloromethane were added to dried samples. The solutions were sonicated for 5 min in an ultrasonic bath and the dispersion was immediately deposited on polycrystalline gold (about 100 nm thick) evaporated on mica. After the deposition, the samples were dried under nitrogen and annealed at 80 °C to remove the solvent and promote the sample adhesion to the substrate. XPS analysis was performed as detailed reported in the ESI.†

### Fabrication of the electrodes array and sensitive films

An aluminum mask was employed in the thermal evaporation (Angstrom Engineering) of 14 gold electrode arrays (1 mm gap) on microscope glass slides (VWR) that had been previously washed in acetone and dried. A 10 nm layer of chromium was deposited first to allow the subsequent adhesion of 100 nm of gold. A quantity of 2 mg of pristine SWCNTs (p-SWCNTs) or functionalized carbon nanotubes (f-SWCNTs) was dispersed in 4 mL of *o*-DCB by sonication in an ultrasonic bath for 1–2 min at room temperature. The resulting dispersion was drop-cast onto the electrodes and dried under vacuum to remove the solvent. Typically, the deposition of 3–5 μL drops was necessary to obtain the target resistance of 100–150 kΩ, checked by a digital multimeter. The 14-electrode array was used to test different materials under identical conditions.

### Delivering system of gases on the device

The functionalized electrodes were placed in a flow chamber constructed from PTFE connected to a gas mixing and delivery system. This system consisted of two digital mass flow controllers to control the flow (0.5–4 mL min^−1^) of NH_3_ or TMA in nitrogen (1% NH_3_ in N_2_ and 1% TMA in N_2_ custom-ordered from Airgas) and to dilute the target gas with N_2_ (0.5–1 L min^−1^) or air (1 L min^−1^).

The analytes were delivered to the device at various concentrations (5–40 ppm) for steps of 100 s. For controlled humidity measurements, the gas mixture was bubbled through water before reaching the PTFE enclosure containing the device. The gas generator system was calibrated for each VOC of interest and used to deliver a known concentration of a given VOC diluted in N_2_ at a fixed gas flow rate to the device's enclosure. Relative humidity was also measured.

### Measurements of device response

The conductivity values from the sensor array were determined by amperometric measurements. For this purpose, the current was measured with the PSTrace software (PalmSens BV) at constant voltage (0.1 V) between the electrodes. In this condition, the conductance (*G* = current/voltage) is directly proportional to current. To correct for differences between device resistances, the conductance is normalized such that Δ*G*/*G*_0_ = (*G*_0_ − *G*)/*G*_0_, where *G*_0_ is the conductance before exposure to NH_3_ or TMA and *G* is the conductance achieved during exposure. In our work, the conductance decreased with analyte exposure and Δ*G*/*G*_0_ was positive. We report the responses as the arithmetic mean of the three replicated sensors for each material.

## Results and discussion

### SWCNTs functionalization and characterization

**Fig. 1:**
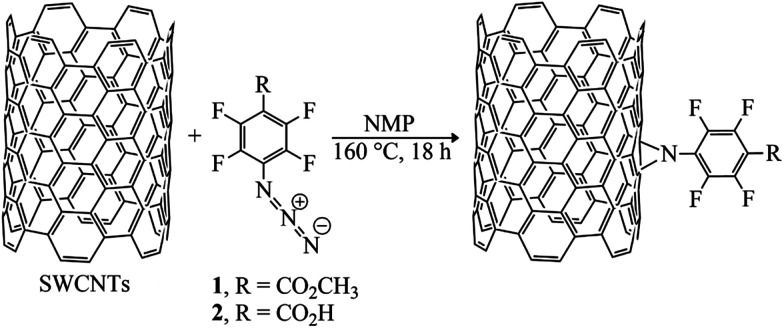
Schematic reaction of SWCNTs with methyl-4-azido-2,3,5,6-tetrafluorobenzoate (1) and 4-azido-2,3,5,6-tetrafluorobenzoic acid (2) *via* nitrene addition.

The azido group served as precursor to generate a highly reactive nitrene intermediate. Herein, the aryl nitrenes were formed by thermal treatment at 160 °C of methyl 4-azido-2,3,5,6-tetrafluorobenzoate (1) and 4-azido-2,3,5,6-tetrafluorobenzoic acid (2) in NMP. This treatment allowed carboxylic acid and methyl ester moieties to be inserted on SWCNTs sidewalls (Fig. 1 and Table 1). Pentafluorophenyl compounds were used since their presence was supposed to foster the interaction of the sensitive material with the target analytes.

**Table 1:** Chemical structure and name of the sensitive compounds synthetised by nitrene chemistry

Name	SWCNT-N-C_6_F_4_CO_2_H	SWCNT-N-C_6_F_4_CO_2_CH_3_
Sensitive compound	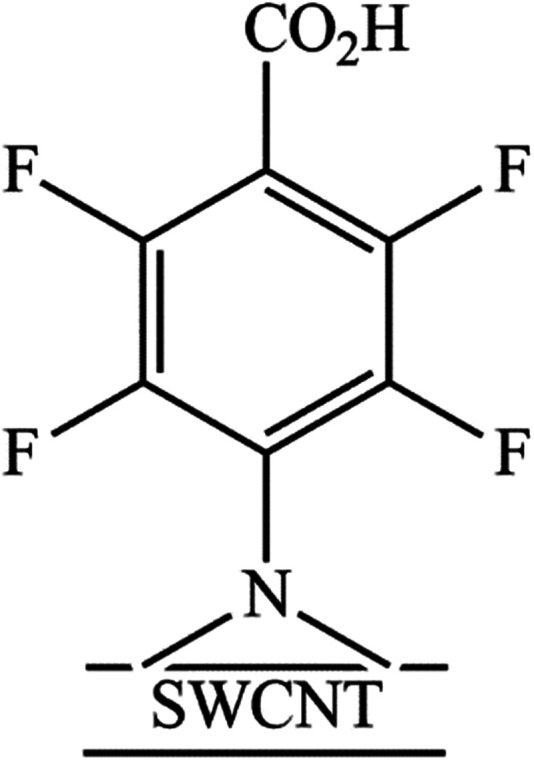	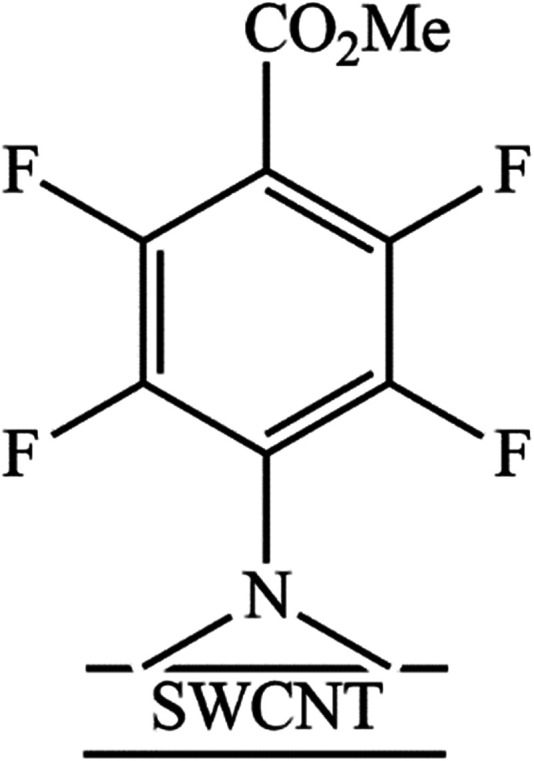

#### XPS analysis

The degree of functionalization of the modified SWCNTs was evaluated by XPS analysis (see ESI†). The comparison of survey spectra confirmed that in the p-SWCNTs sample only carbon and oxygen are present (excluding the signals due to the gold substrate), whereas nitrogen and fluorine were detected in functionalized samples (Table 2). In good agreement with previous reports,^25^ the C 1s region is characterized by 6 components: C=C (284.53–284.38 eV), C–C (285.5–285.11 eV), C–O/C–N (287.53–286.21 eV, our experimental setup did not allow these two components to be discriminated), C=O (287.92–286.45 eV), O–C=O (289.54–288.39 eV), and π–π (this component can be attributed to the delocalization of the electron on the surface of the nanotube). The C–F contribution was not found because of the interference with the π–π peak. Absorbed CO and CO_2_ within the porous structure of p-SWCNTs tubes possibly contributed to the carbonyl structure of the carbon peak.^26^

**Table 2:** Components in p-SWCNTS and f-SWCNTs as determined by XPS analysis

Sample	Component	Peak position (eV)	FWHM (eV)	Sensitivity	Corrected area	Percentage
Pristine SWCNTs	*F*	*—*	*—*	*1*	*0*	*0.0%*
	*O*	*531.8*	*3.1*	*0.7*	*408*	*4.3%*
	*N*	*—*	*—*	*0.5*	*0*	*0.0%*
	C=C	284.5	1.1	0.3	5963	63.2%
	C–C	285.5	1.1	0.3	1297	13.7%
	C–O/C–N	286.4	1.1	0.3	645	6.8%
	C=O	287.6	1.1	0.3	453	4.8%
	COO	288.9	1.1	0.3	338	3.6%
	π–π	290.5	1.1	0.3	338	3.6%
	*C total*	*—*	*—*	*0.3*	*9034*	*95.7%*
SWCNTs-CO_2_CH_3_	*F*	*687.8*	*2.2*	*1*	*336*	*1.7%*
	O=C	531.6	1.9	0.7	1514	7.7%
	O–C	533.2	1.9	0.7	523	2.7%
	*O total*	*—*	*—*	*0.7*	*2037*	*10.3%*
	*N*	*400.3*	*1.8*	*0.5*	*1323*	*6.7%*
	C=C	284.5	1.1	0.3	6892	34.9%
	C–C	285.3	1.1	0.3	3240	16.4%
	C–O/C–N	286.2	1.1	0.3	2622	13.3%
	C=O	287.3	1.1	0.3	1503	7.6%
	COO	288.4	1.1	v	1179	6.0%
	π–π	289.8	1.1	0.3	598	3.0%
	*C total*	*—*	*—*	*0.3*	*16 034*	*81.3%*
SWCNTs-CO_2_H	*F*	*687.9*	*2.0*	*1*	*231*	*0.9%*
	O=C	531.6	2.1	0.7	1941	7.8%
	O–C	533.5	2.1	0.7	650	2.6%
	*O total*	*—*	*—*	*0.7*	*2591*	*10.4%*
	*N*	*400.3*	*1.9*	*0.5*	*1618*	*6.5%*
	C=C	284.5	1.1	0.3	8936	35.8%
	C–C	285.3	1.1	0.3	4236	17.0%
	C–O/C–N	286.3	1.1	0.3	3385	13.6%
	C=O	287.4	1.1	0.3	1922	7.7%
	COO	288.6	1.1	0.3	1324	5.3%
	π–π	290.1	1.1	0.3	686	2.8%
	*C total*	*—*	*—*	*0.3*	*20 490*	*82.2%*

**Fig. 2:**
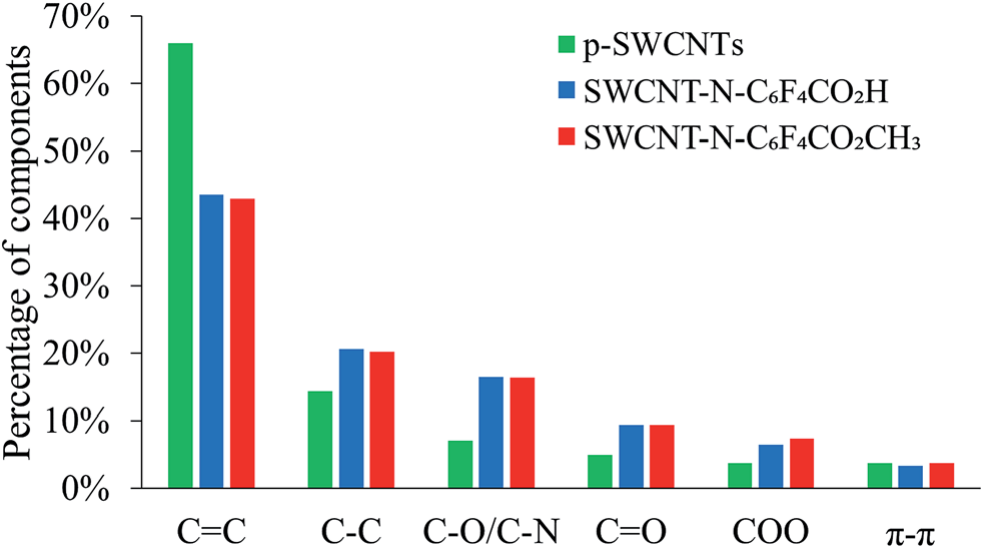
Relative percentages of the components found in the C 1s region in p-SWCNTs and f-SWCNTs after XPS analysis.

Fig. 2 shows the relative percentages of each component. After the functionalization, the C=C component dramatically decreased and the C–C and oxygenated components increased. The COO component was higher in the SWCNT-N-C_6_F_4_CO_2_CH_3_ than in the SWCNT-N-C_6_F_4_CO_2_H. This difference can be attributed to the higher functionalization ratio of SWCNT-N-C_6_F_4_CO_2_CH_3_, which was also confirmed from the area of fluorine peak.

Table 2 shows that in p-SWCNTs the percentages of F 1s and N 1s were 0%, whereas there was a very low percentage of oxygen (4.3%). In p-SWCNTs, the ratio between the component C=C and C–C was about 4.5. These data confirmed that the oxidation of the p-SWCNTs sample was very low with a high unsaturation degree. In SWCNT-N-C_6_F_4_CO_2_CH_3_ and SWCNT-N-C_6_F_4_CO_2_H, the percentages of F 1s were 1.7% and 0.9%, respectively, whereas the ratio between the components C=C and C–C decreased to about 2. In f-SWCNTs, the oxygen peak was higher than in p-SWCNTs. This result allowed the components O=C and O–C to be discriminated. Notably, the O=C component had an equal percentage of the component C=O, which confirmed the fitting goodness. The fitting goodness could not be determined for the O–C component, which could not be distinguished from the C–N component because of a similar binding energy.^25^

The N 1s percentage was high when compared with the F 1s percentage (every functional group had 1 atom of nitrogen and 4 atoms of fluorine). This result can be explained by a nitrogen contamination probably due to the residue of the solvent used in the functionalization reaction (*N*-methyl-2-pyrrolidone: NC_5_O). This residue affected not only the nitrogen area but also the carbon area. Therefore, using the area of F 1s to calculate the expected area of nitrogen associated with the functional groups, we corrected the carbon area to eliminate the solvent contribution. The functionalization ratio for the SWCNT-N-C_6_F_4_CO_2_H sample was 1 functional group every 213 carbon atoms of SWCNTs and in the SWCNT-N-C_6_F_4_CO_2_CH_3_ is 1 functional group every 109 carbon atoms of SWCNTs.

#### Raman spectroscopy

**Fig. 3:**
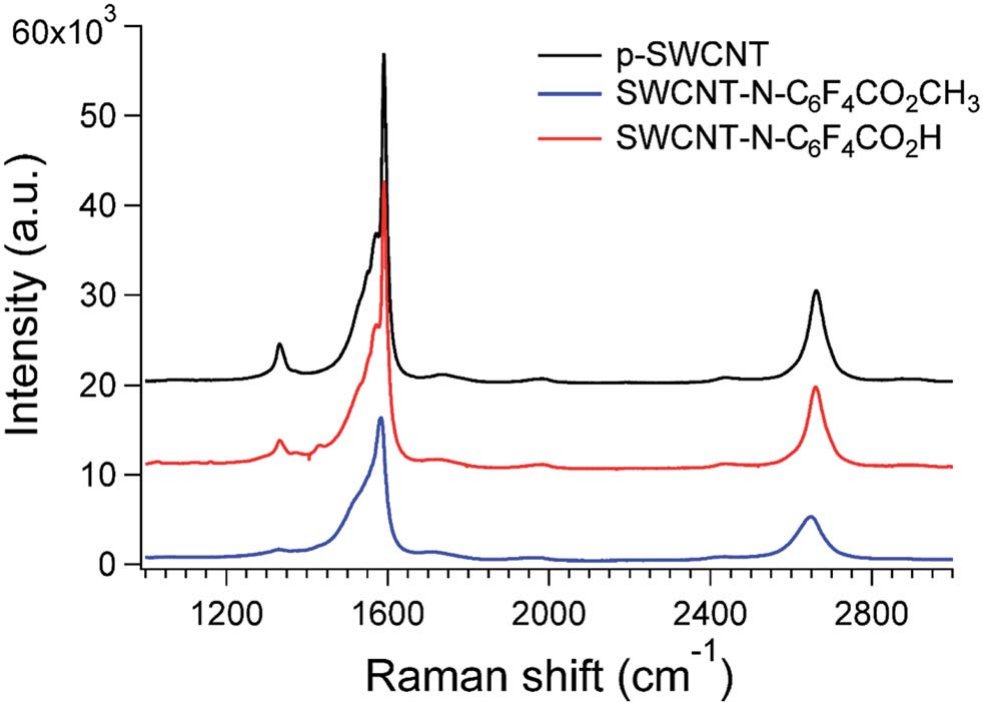
Raman spectra (532 nm excitation) of p- and f-SWCNTs.

In Raman spectroscopy, the G-band (at about 1590 cm^−1^) is associated with the ordered sp^2^ hybridized carbon structure typical of graphite, whereas the D-band (at about 1330 cm^−1^) is due to the amorphous carbon and local defects that originate from structural imperfections.^27^ The general trend shows that after CNTs covalent functionalization, the G/D ratio decreases and the width of the D-band increases.^28^Fig. 3 shows the Raman spectra for p-SWCNTs and f-SWCNTs and in Table 3 the modes and intensity peaks of D and G bands are reported. Notably, because of the functionalization process, the intensity ratio of the D- and G-bands (*I*_D_/*I*_G_) dropped over 50% whereas the full width at half maximum (FWHM) increased importantly. This contradictory phenomenon can be addressed to the opposite effects of the removal of impurities and the creation of defects because of the functionalization.^26^ Moreover, the opposite trend of the *I*_D_/*I*_G_ ratio can be also supported considering a recently published [1,2] cycloaddition of CNTs by means of electron-poor aromatic nitrenes.^29^ In that work, the authors demonstrated that after the cycloaddition step, subsequent rehybridization restores the sp^2^ state, thus recovering the aromaticity of the system in the defected region of CNTs. Since this mechanism is found for electron deficient aromatic derivatives, a similar explanation can be also provided using azido-tetrafluorobenzoic derivatives as functionalizing agents.

**Table 3:** Spectral features for D and G bands derived from Raman spectroscopy for p- and f-SWCNTs

Sample	D peak (cm^−1^)	G peak (cm^−1^)	*I*_D_/*I*_G_	D band FWHM (cm^−1^)
p-SWCNTs	1333.2 ± 0.6	1591.3 ± 0.0	0.12 ± 0.01	30.5 ± 1.8
SWCNT-N-C_6_F_4_CO_2_H	1335.7 ± 1.5	1591.9 ± 0.0	0.06 ± 0.0	136.8 ± 5.0
SWCNT-N-C_6_F_4_CO_2_CH_3_	1328.2 ± 1.6	1584.2 ± 0.2	0.04 ± 0.01	56.8 ± 9.0

#### SEM analysis

**Fig. 4:**
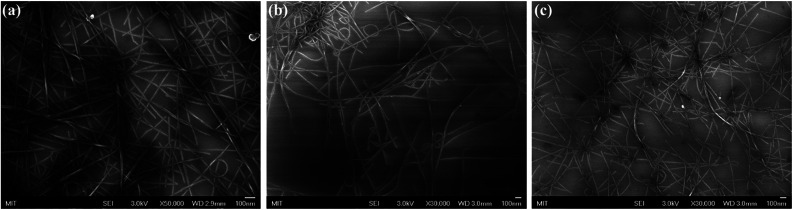
Representative SEM images of (a) p-SWCNTs, (b) SWCNT-N-C_6_F_4_CO_2_CH_3_ and (c) SWCNT-N-C_6_F_4_CO_2_H samples. Typical accelerating voltages were 3.0 kV.

SEM analysis revealed the high density of well-exfoliated f-SWCNTs, which were not shortened or broken by the functionalization procedure (Fig. 4). This result confirms the effectiveness of the nitrene chemistry in providing undamaged functionalized CNTs thank to its mild reaction conditions.^22,23^

### Ammonia and trimethylamine gas sensing tests

**Fig. 5:**
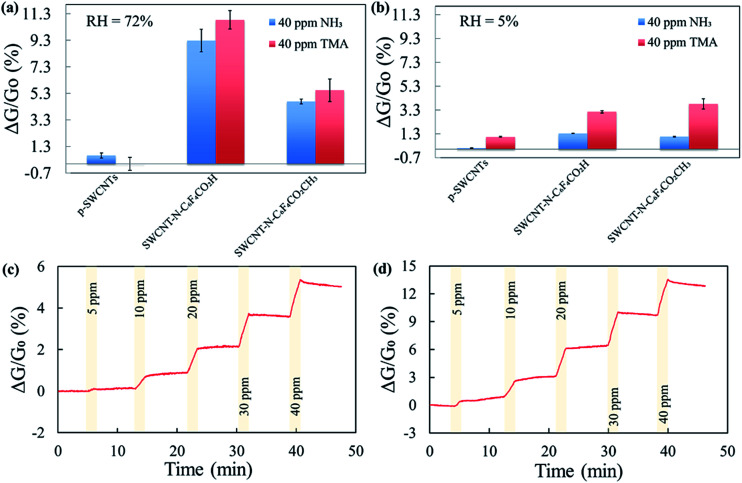
Example of the responses of pristine and functionalized SWCNTs at different humidity (RH%) conditions to exposures of 40 ppm of NH_3_ and TMA in (a) N_2_ at RH of 72% and (b) in air at RH < 5%; percentages of the conductance changes in N_2_ (<5% RH) of SWCNT-N-C_6_F_4_CO_2_H in response to increasing concentrations of (c) NH_3_ and (d) TMA. The exposure time was 100 s. A linear fit of the baseline was subtracted from the data.

At room temperature, all sensors exhibited a maximum saturation response towards NH_3_ and TMA at 40 ppm. The response of SWCNTs after exposure to 40 ppm of NH_3_ and TMA was assessed in N_2_ at controlled humidity conditions (three replicates per each of the CNTs). At relative humidity (RH) of 72%, SWCNT-N-C_6_F_4_CO_2_H was more responsive (Δ*G*/*G*_0_ (%) = 10.8 ± 0.7 and 9.3 ± 0.8, for NH_3_ and TMA respectively) than SWCNT-N-C_6_F_4_CO_2_CH_3_ (Δ*G*/*G*_0_ (%) = 4.7 ± 0.2 and 5.5 ± 0.8, for NH_3_ and TMA respectively), whereas p-SWCNT had a negligible conductance variation (Fig. 5a). Our sensors also have good performance in air with similar variations, although with slightly lower responses (Fig. 5b).

The difference among f-SWCNTs is likely related to the strong Brønsted acid/base interaction of the carboxylic acid moiety with the lone-pair of the electron donor NH_3_ and TMA. Therefore, SWCNT-N-C_6_F_4_CO_2_H was the best candidate for the realization of NH_3_/TMA gas sensors, thus it was subject to further investigation. Fig. 5c and d show the response of three replicate sensors exposed to 5, 10, and 40 ppm of NH_3_ and TMA for 100 s each in air (<5% RH), respectively (3 exposures for each concentration). The sensor response time was about 2 min.

The decrease of conductance upon exposure can be explained considering the electronic nature of SWCNTs and their mechanisms of charge transfer. In the p-type semiconducting SWCNTs, the interaction with the donor molecules NH_3_ and TMA decreases the conductance of the network since the charge transfer from the amines effectively refills the holes in the valence band.^30^ This explains why p-SWCNTs are also sensitive to NH_3_ and TMA. The modification of the SWCNTs surface improves the interactions of the SWCNTs and enhances the electrical response.

**Fig. 6:**
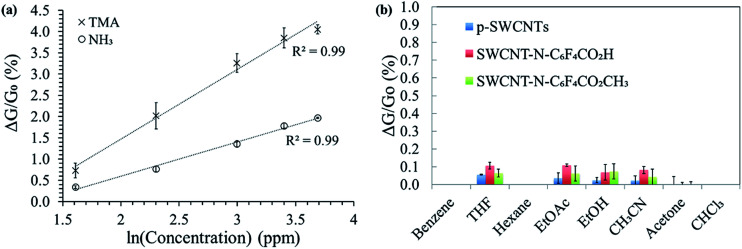
(a) Response of SWCNT-N-C_6_F_4_CO_2_H at a single exposure of various ppm concentrations (expressed as natural logarithm) of NH_3_ and TMA in air. (Dotted line: linear fit *y* = *a* + *b × x*. TMA: *a* = −1.812 ± 0.281 and *b* = 1.641 ± 0.097; NH_3_: *a* = −1.014 ± 0.113 and *b* = 0.807 ± 0.039); (b) dependence of the p-SWCNTs and f-SWCNTs from several VOCs.

Fig. 6a shows the calibration curves of SWCNT-N-C_6_F_4_CO_2_H in response to 100 s exposure of NH_3_ and TMA over the range of 5–40 ppm. The limit of detection was found to be 0.2 ppm, a value that placed the designed device at the topmost positions in amine sensing based on SWCNTs.^31^ The enhanced sensitivity was ascribed to the functionalization process that also contributes in removal of loose SWCNTs agglomerates with poor electrical contact and a more efficient transport of carriers. Moreover, our design involved a more simple but effective functional probes with respect to those reported in the recent literature and based on combination of Au nanoparticles or functionalized polyanilines.^31^

A typical linear behaviour is gathered from semi-log plots. The higher sensitivity of the sensor towards the detection of TMA relative to NH_3_ can be ascribed to its more basic nature, indicating a more favoured lone electron pair interaction with SWCNTs and the pendant N-C_6_F_4_CO_2_H groups.^32^ We observed a saturation above 40 ppm (not shown) that was most likely due to a strong interaction between the gas molecules and SWCNT materials.

We eventually checked the interference of other gases by observing the response of the sensor devices based on pristine and functionalized SWCNTs to a wide range of volatile organic compounds (VOCs) such as: benzene, tetrahydrofurane (THF), hexane, ethyl acetate (AcOEt), ethanol, acetonitrile (CH_3_CN), acetone and chloroform (CHCl_3_). Very low interferences were observed from all the organic vapours investigated with Δ*G*/*G*_0_ (%) values lower than 0.1 at 200 ppm of VOCs (three replicates per each of the CNTs, Fig. 6b), indicating the high selectivity of the sensors toward NH_3_ and TMA.

When NH_3_ or TMA interacts with the perfluorinated functionalizing molecule, a change organic molecular component to an anion can also lead to depletion or trapping of the holes of CNTs to reduce the system conductance.

## Conclusions

This work described the preparation of a chemiresistive sensor based on sidewall modified SWCNTs for the detection of low concentrations (5–40 ppm) of gaseous NH_3_ and TMA at room temperature. The XPS analysis confirmed the effectiveness of the nitrene-based functionalization in efforting 1 functional group every 213 and 109 carbon atoms for SWCNT-N-C_6_F_4_CO_2_H and SWCNT-N-C_6_F_4_CO_2_CH_3_, respectively. Notably, f-SWCNTs were demonstrated to be at least 2-fold more sensitive than p-SWCNTs. It was worth noting that SWCNT-N-C_6_F_4_CO_2_H-based sensor was capable of discriminating the amount of NH_3_ and TMA emitted up to 40 ppm. Notably, the sensor remained responsive at high humidity, in the presence of air and showed no interference from all other gases of volatile organic compounds investigated, *i.e.* Δ*G*/*G*_0_ (%) < 0.1 at 200 ppm of VOCs. Efforts are being made to improve the output range and achieve a better discrimination between different volatile molecules. Nevertheless, this sensor is ready as a single-use indicator of the threshold crossing, about 10 ppm, of TMA and NH_3_ concentrations to monitor the freshness of packaged seafood products. Future combination with a RFID tag can lead to fast real-time seafood intelligent packaging.

## Conflicts of interest

There are no conflicts to declare.

## Supplementary Material

RA-008-C7RA13304A-s001
