# Supplementary material for: Room temperature amine sensors enabled by sidewall functionalization of single-walled carbon nanotubes
Source: RSC Adv. 2018 Feb 1;8(10):5578–85. doi: 10.1039/c7ra13304a (PMC6390973; doi:10.1039/c7ra13304a)
Supplement: RA-008-C7RA13304A-s001 [file RA-008-C7RA13304A-s001.pdf]

Electronic Supplementary Material (ESI) for RSC Advances.
This journal is © The Royal Society of Chemistry 2018

Supporting information

Room temperature amine sensors enabled by sidewall functionalization of single-walled carbon nanotubes

Synthesis of methyl 4-azido-2,3,5,6-tetrafluorobenzoate (1)

Methyl 4-azido-2,3,5,6-tetrafluorobenzoate (**1**) was synthesized according to the methods described by Keana et al.[25] Methyl pentafluorobenzoate (10.0 g, 44.2 mmol) and NaN_3 (3.00 g, 46.1 mmol) were dissolved in a 3:1 (v/v) mixture of acetone and water (30 mL) and refluxed for 8 h at 85 °C. Since sodium azide can be explosive when heated as a solid and is highly toxic, this procedure must be done in a fume hood with an additional blast shield in place. The mixture was then cooled to room temperature, diluted with H_2O (250 mL) and extracted with Et_2O (3×300 mL). The combined organic layers were dried over $MgSO_4$ and the solvent was removed under reduced pressure. Drying under vacuum overnight afforded (**1**) as a white solid (8.84 g, 80% yield). ^1H NMR ($CDCl_3$): δ 3.99 (s, 3 H). ^{19}F NMR ($CDCl_3$): δ –138.6 (m, 2 F), –150.9 (m, 2 F). HRMS m/z: [$M+H^+$] calcd for $C_8H_3F_4N_3O_2$: 250.0234, found: 250.0237.

Synthesis of methyl 4-azido-2,3,5,6-tetrafluorobenzoic acid (2)

4.00 g of (**1**) (16.1 mmol) were treated with 20% aqueous NaOH (5.3 mL) in MeOH (66.7 mL) and H_2O (7.0 mL). The mixture was stirred at room temperature for 24 h. The solution was acidified with 2N HCl in an ice bath to pH < 1 and extracted by $CHCl_3$ (3×100 mL). The combined organic layers were dried over $MgSO_4$ and the solvent was concentrated under reduced pressure. The product 4-azido-2,3,5,6-tetrafluorobenzoic acid (**2**) was obtained as a white solid (3.42 g, yield 91%). ^{19}F NMR ($CDCl_3$): δ –137.1 (m, 2 F), –150.7 (m, 2 F). HRMS m/z: [$M–H^–$] calculated for $C_7H_2F_4N_3O_2$: 233.9932, found: 233.9919.

XPS analysys

Briefly, the samples were fixed on a sample holder that was installed in an ultra-high vacuum (UHV) analysis chamber (pressure $5•10^{-8}$ mbar). The X-ray non-monochromatic source (Mg Kα radiation, 1253.6 eV) was operated at 100 W (10 kV and 10 mA). XPS spectra were measured at normal emission with a fixed pass energy of 44 eV and 22 eV for survey and high-resolution spectra, respectively. The inelastic background in the spectra was subtracted by Shirley's method.[1] The spectra were aligned by placing the component of the

Au 4f7/2 spectra at 84.0 eV in good agreement with earlier reports.[2] Semi-quantitative analysis has been carried out estimating the intensity of each component using CasaXPS software (Casa Software Ltd., UK) and correcting the extracted areas using the atomic sensitivity factors.[3]

XPS Spectra

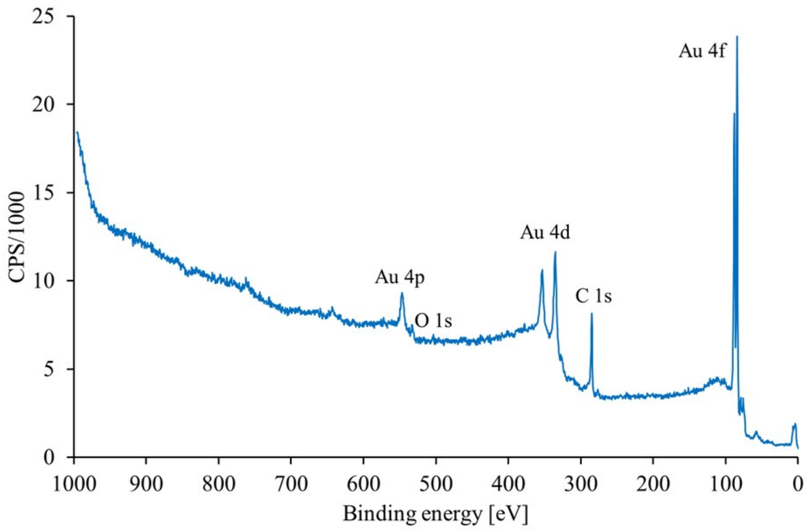

Figure S1. XPS spectrum of *p*-SWCNTs.

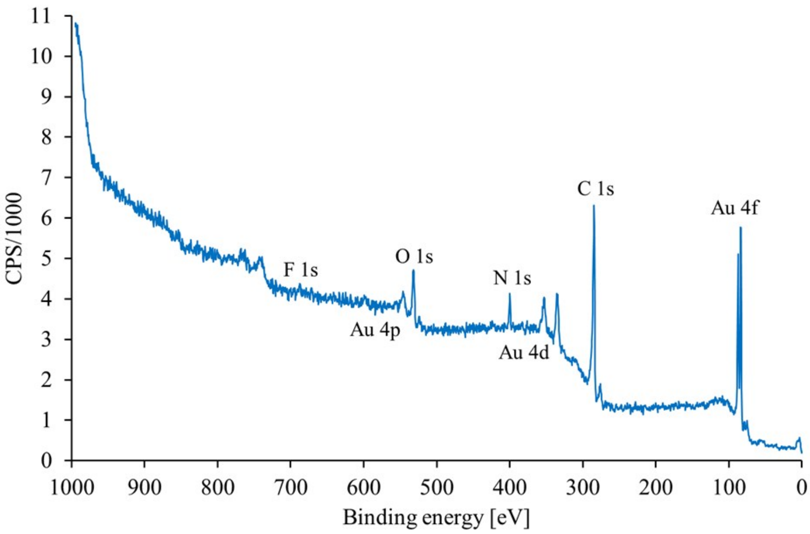

Figure S2. XPS spectrum of SWCNT-N-$C_6F_4CO_2H$.

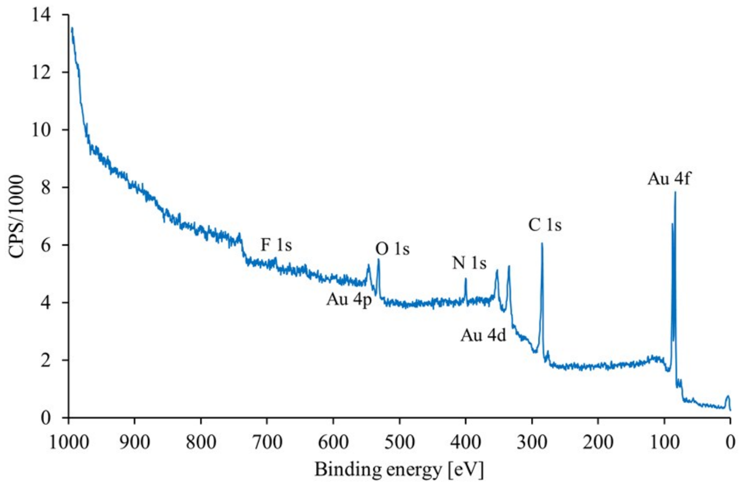

Figure S3. XPS spectrum of SWCNT-N-$C_6F_4CO_2CH_3$.

XPS: fitting

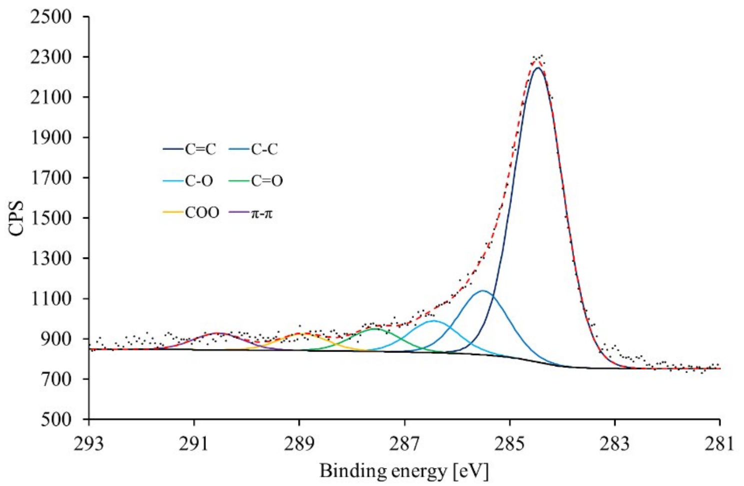

Figure S4. Fitting of the C1*s* region for *p*-SWCNTs.

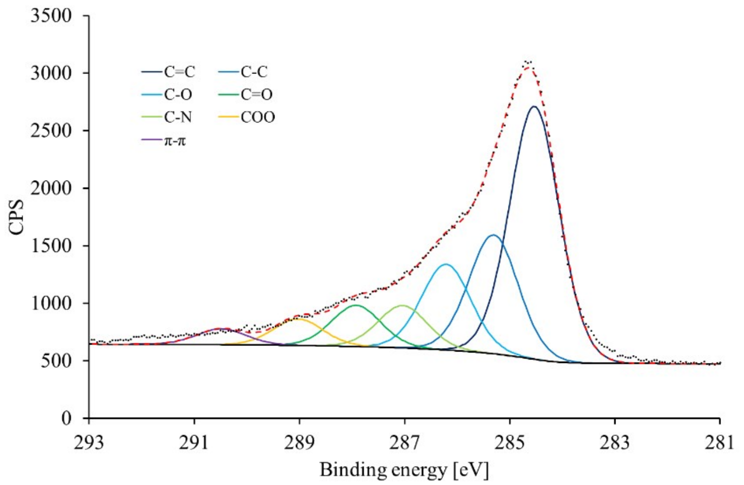

Figure S5. Fitting of the C1*s* region for SWCNT-N-$C_6F_4CO_2H$.

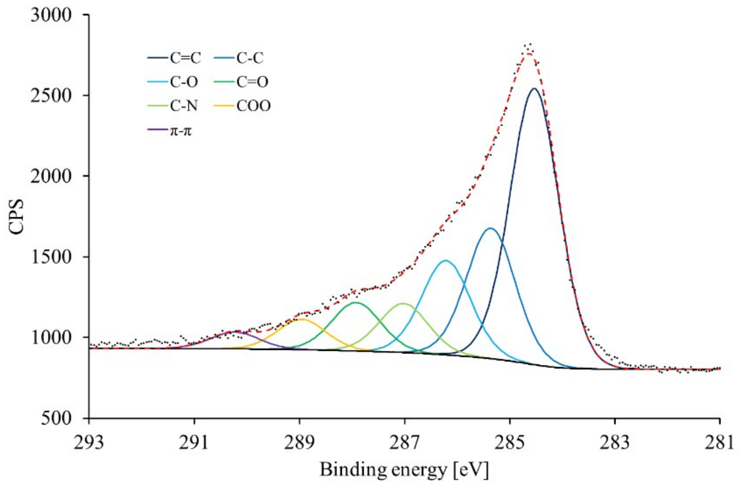

Figure S6. Fitting of the C1*s* region for SWCNT-N-$C_6F_4CO_2CH_3$.

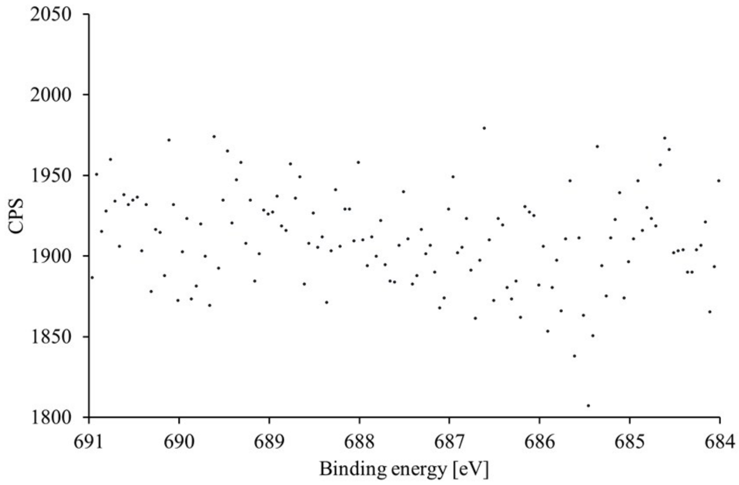

Figure S7. Fitting of the F 1*s* region for *p*-SWCNTs.

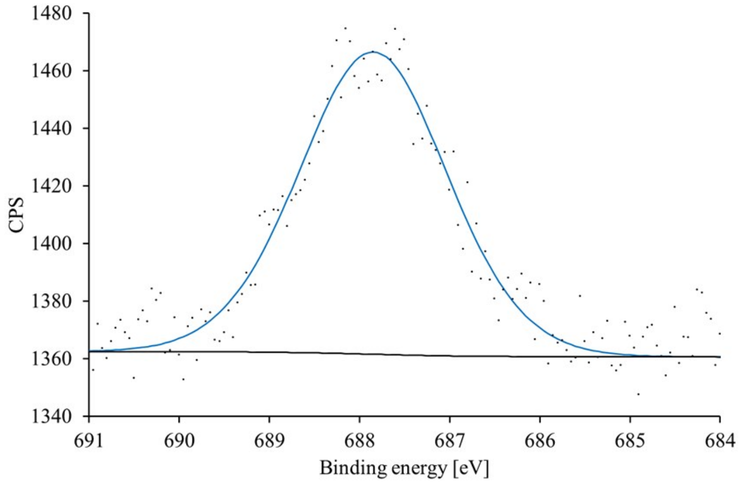

Figure S8. Fitting of the F 1s region for SWCNT-N-$C_6F_4CO_2H$.

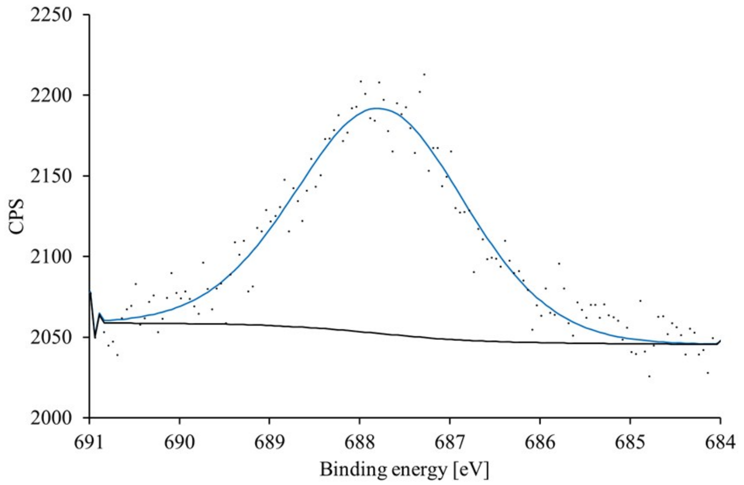

Figure S9. Fitting of the F 1*s* region for SWCNT-N-$C_6F_4CO_2CH_3$.

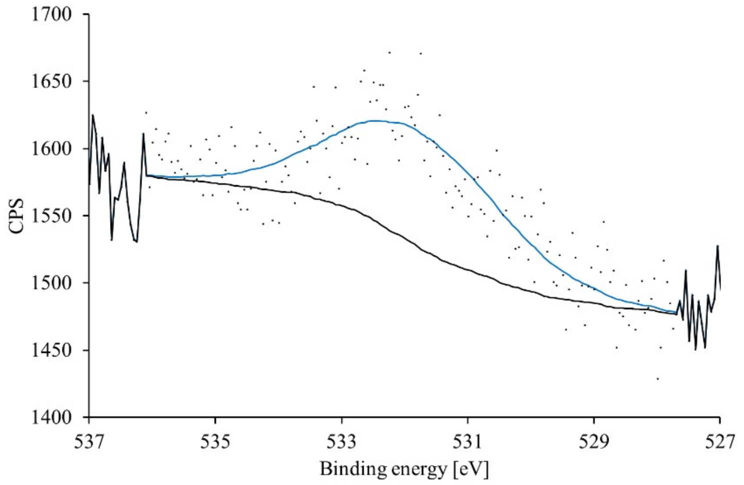

Figure S10. Fitting of the O 1s region for *p*-SWCNTs.

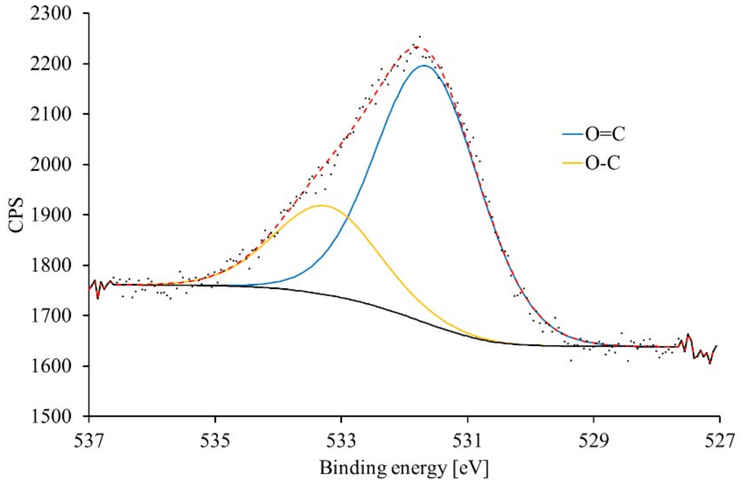

Figure S11. Fitting of the O 1s region for SWCNT-N-$C_6F_4CO_2H$.

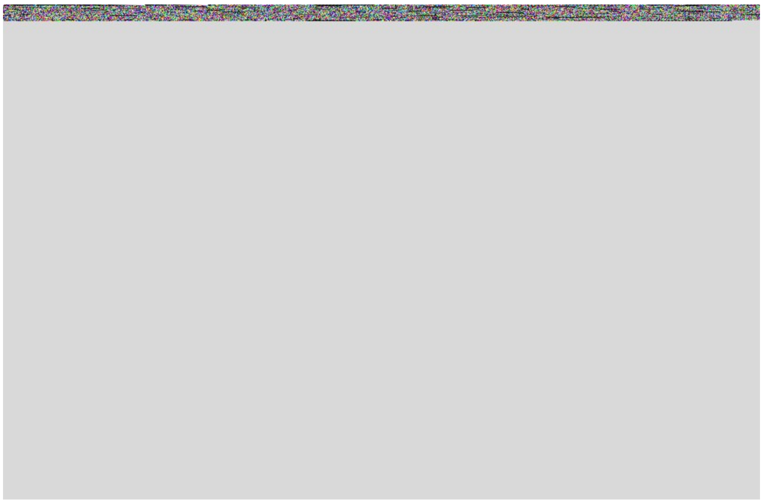

Figure S12. Fitting of the O 1*s* region for SWCNT-N-$C_6F_4CO_2CH_3$.

References

1. D. A. Shirley, *Physical Review B*, 1972, **5**, 4709-4714.
2. J. Radnik, C. Mohr and P. Claus, *Physical Chemistry Chemical Physics*, 2003, **5**, 172-177.
3. D. Briggs, *Surface and Interface Analysis*, 1981, **3**, v-v.
